# Spatially Pooled Contrast Responses Predict Neural and Perceptual Similarity of Naturalistic Image Categories

**DOI:** 10.1371/journal.pcbi.1002726

**Published:** 2012-10-18

**Authors:** Iris I. A. Groen, Sennay Ghebreab, Victor A. F. Lamme, H. Steven Scholte

**Affiliations:** 1 Cognitive Neuroscience Group, Department of Psychology, University of Amsterdam, Amsterdam, The Netherlands; 2 Intelligent Systems Lab Amsterdam, Institute of Informatics, University of Amsterdam, Amsterdam, The Netherlands; Indiana University, United States of America

## Abstract

The visual world is complex and continuously changing. Yet, our brain transforms patterns of light falling on our retina into a coherent percept within a few hundred milliseconds. Possibly, low-level neural responses already carry substantial information to facilitate rapid characterization of the visual input. Here, we computationally estimated low-level contrast responses to computer-generated naturalistic images, and tested whether spatial pooling of these responses could predict image similarity at the neural and behavioral level. Using EEG, we show that statistics derived from pooled responses explain a large amount of variance between single-image evoked potentials (ERPs) in individual subjects. Dissimilarity analysis on multi-electrode ERPs demonstrated that large differences between images in pooled response statistics are predictive of more dissimilar patterns of evoked activity, whereas images with little difference in statistics give rise to highly similar evoked activity patterns. In a separate behavioral experiment, images with large differences in statistics were judged as different categories, whereas images with little differences were confused. These findings suggest that statistics derived from low-level contrast responses can be extracted in early visual processing and can be relevant for rapid judgment of visual similarity. We compared our results with two other, well- known contrast statistics: Fourier power spectra and higher-order properties of contrast distributions (skewness and kurtosis). Interestingly, whereas these statistics allow for accurate image categorization, they do not predict ERP response patterns or behavioral categorization confusions. These converging computational, neural and behavioral results suggest that statistics of pooled contrast responses contain information that corresponds with perceived visual similarity in a rapid, low-level categorization task.

## Author Summary

Humans excel in rapid and accurate processing of visual scenes. However, it is unclear which computations allow the visual system to convert light hitting the retina into a coherent representation of visual input in a rapid and efficient way. Here we used simple, computer-generated image categories with similar low-level structure as natural scenes to test whether a model of early integration of low-level information can predict perceived category similarity. Specifically, we show that summarized (*spatially pooled*) responses of model neurons covering the entire visual field (*the population response*) to low-level properties of visual input (*contrasts*) can already be informative about differences in early visual evoked activity as well as behavioral confusions of these categories. These results suggest that low-level population responses can carry relevant information to estimate similarity of controlled images, and put forward the exciting hypothesis that the visual system may exploit these responses to rapidly process real natural scenes. We propose that the spatial pooling that allows for the extraction of this information may be a plausible first step in extracting scene gist to form a rapid impression of the visual input.

## Introduction

Complex natural images are categorized remarkably fast [1], [2], sometimes even faster than simple artificial stimuli [3]. For animal and non-animal scenes, differences in EEG responses are found within 150 ms [4] and a correct saccade is made within 120 ms [5]. This speed of processing is also found for other scene categories [6] and may require less attentional resources compared to artificial images [7], [8]. This suggests that relevant visual information is rapidly and efficiently extracted from early visual responses to natural scenes. However, the neural computations involved in this process are not known.

Importantly, natural images differ from other image types such as white noise in low-level properties (e.g., sparseness), leading to the suggestion that the visual system has adapted to these low-level properties [9]. This idea paved the way for optimal coding models for natural images [10], [11] and successful predictions of response properties of visual neurons [12]. Recent work identified statistical properties that differ even within the class of natural images, e.g. between natural scene parts [13], [14] or natural image categories [15], showing that image statistics such as power spectra of spatial frequency content or distributions of local image features are informative about scene category.

**Figure 1:**
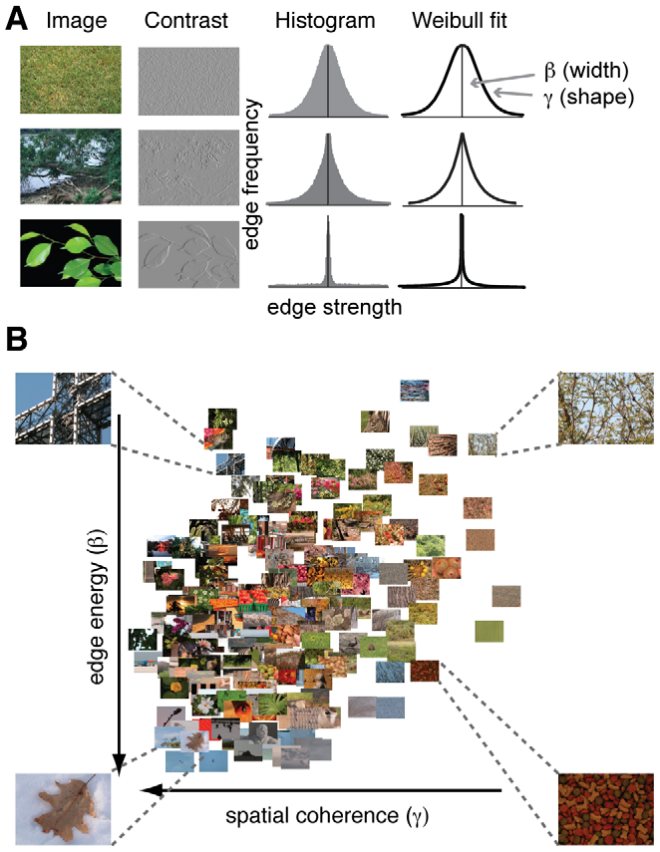
Contrast histograms of natural images follow a Weibull distribution. (**A**), Three natural images with varying degrees of details and scene fragmentation. The homogenous, texture-like image of grass (upper row) contains many edges of various strengths; its contrast distribution approaches a Gaussian. The strongly segmented image of green leaves against a uniform background (bottom row) contains very few, strong edges that are highly coherent; its distribution approaches power law. Most natural images, however, have distributions in between (middle row). The degree to which images vary between these two extremes is reflected in the free parameters of a Weibull fit to the contrast histogram: β (beta) and γ (gamma). (**B**), For each of 200 natural scenes, the beta and gamma values were derived from fitting the Weibull distribution to their contrast histogram. Beta describes the width of the histogram: it varies with the distribution of local contrasts strengths. Gamma describes the shape of the histogram: it varies with the amount of scene clutter. Four representative pictures are shown in each corner of the parameter space. Images with a high degree of scene segmentation, e.g. a leaf on top of snow, are found in the lower left corner, whereas highly cluttered images are on the right. Images with more depth are located on the top, whereas flat images are found at the bottom. Images are from the McGill Calibrated Colour Image Database [86].

The fact that it is mathematically possible to distinguish categories based on image statistics, however, does not imply that they are used for categorization in the brain. Image statistics may not be sufficiently reliable, or their computation may not be suitable for neural implementation [12], [16]. We recently showed that statistics derived from the frequency histogram of local contrast – summarized by two parameters of a Weibull fit, **Fig. 1A** – explain up to 50% of the variance of event-related potentials (ERPs) recorded from visual cortex [17]. These parameters inform about the width and shape of the histogram, respectively, and appear to describe meaningful variability between images (**Fig. 1B**). Importantly, we found that these parameters can be reliably approximated by linear summation of the output of localized difference-of-Gaussians filters modeled after X- and Y-type LGN cells, suggesting that this global information may be available to visual cortex directly from its early low-level contrast responses [17].

Moreover, we found that output of contrast filters with a larger range of receptive field sizes captures additional image information [18]. This is not surprising since objects in natural scenes appear at many distances and hence spatial scales [19]. In the present implementation, the model first estimates at which scale relevant contrast information is present, as well as characteristics of the distribution of contrast strengths at those scales. This model, which approximates early visual population responses based on spatially pooled contrasts, was able to explain almost 80% of ERP variance to natural images [18].

These previous findings suggest that images with more similar contrast response statistics evoke more similar early visual activity. Could these responses already contain relevant information about the stimulus for rapid categorization? The two parameters appear to index meaningful information such as degree of clutter, depth and figure-ground segmentation [17], but how the two dimensions in **Fig. 1B** influence perception has not been examined. The goal of the current study was thus to explore what type of visual information is contained in the variance of the earliest visual contrast responses that is so well described by these two parameters. Specifically, we were interested in whether these parameters cannot only predict variance in visual activity, but also ‘variance in perception’. In other words, do images with more similar contrast statistics also lead to more similar perceptual representations, and perhaps ultimately, to similar images being considered a single category?

We aimed to answer this question in a data-driven manner, by investigating 1) which images group by similarity early in visual processing and 2) whether this grouping matches with perceived similarity of those images. For the first part of this question, we obtained reliable evoked responses to individual images. The advantage of this approach relative to traditional ERP analysis (which is based on averaging many trials across individual images within an a priori determined condition) is that it provides much richer data [20]–[24] that can be used for model selection. We used these single-image evoked responses to compute dissimilarities in ‘neural space’, similar to the pattern analysis approach used in fMRI [25], [26]. This allowed us to track, over the course of the ERP, to what extent the representation of an image is (dis)similar to all images in the data set.

**Figure 2:**
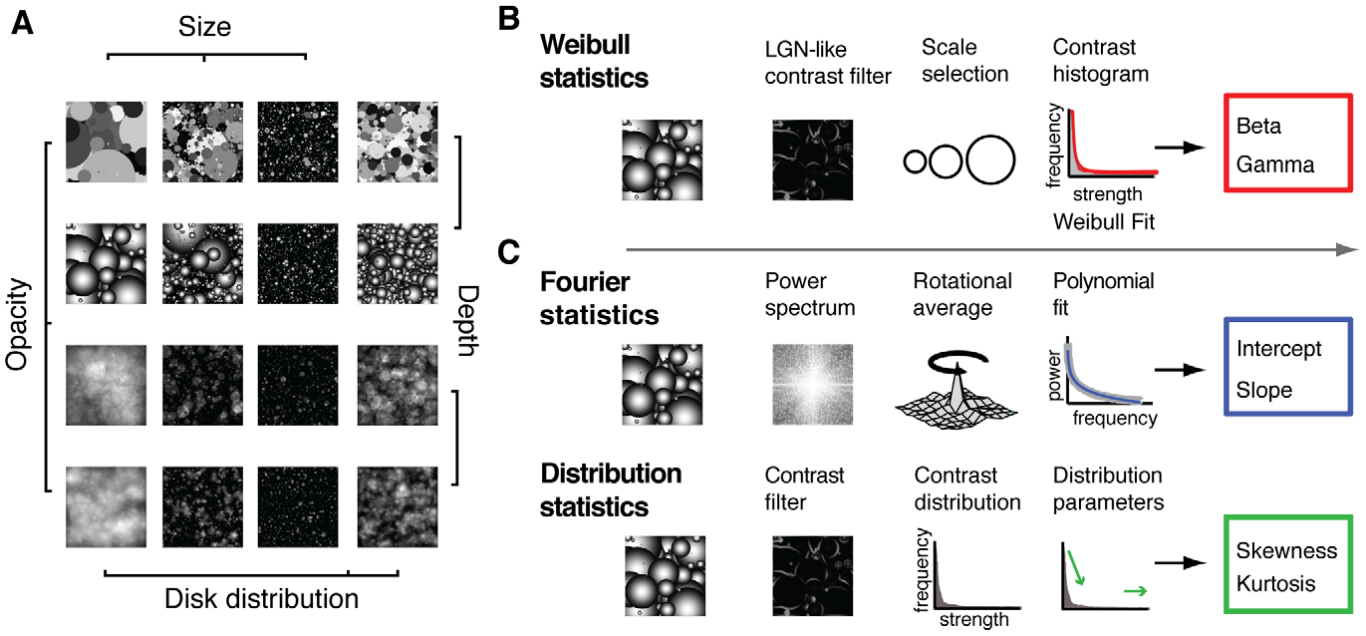
Example stimuli and computation of contrast statistics. (**A**), Example images of each of the 16 categories used in the behavioral and EEG experiment. Images contained randomly placed disks that differed in distribution, opacity, depth and size. Each category contained 16 unique images. (**B**), Consecutive steps in computing various contrast statistics. Weibull statistics are computed by filtering the image with a range of contrast filters with LGN-like scale- and gain properties, after which for each image location, the filter containing the minimal reliable response is selected. Responses of all selected filters are summed in a histogram to which the Weibull function is fitted, from which the beta and gamma parameters are derived using maximum likelihood estimation. (**C**), Power spectra parameters (top row) are extracted by taking the Fourier transform, averaging across directions, and computing the intercept and slope values of a line fitted to the average power spectrum. Higher-order properties of the contrast distribution (bottom row) are computed by filtering with a single-scale center-surround filter, after which skewness and kurtosis of the resulting contrast distribution are derived. Weibull statistics (multiscale local contrast) presumably contain information present in Fourier parameters (scale statistics) as well as local contrast distribution parameters (distribution statistics).

For the second part of the question, we needed to obtain an image-specific behavioral judgment of perceived visual similarity. However, simply judging similarity of natural scenes is problematic, because these images obviously contain rich semantic content: there are many features of natural scenes that can be similar or dissimilar, which is likely to lead to different categorization strategies by different subjects. Also, it is uncertain to what extent specific semantic tags that are provided by the researcher (e.g. ‘openness’ or ‘naturalness’, [27]), can be uniformly interpreted as a relevant stimulus dimension that has a linear mapping to processing in early vision. Therefore, to explore the variance explained by contrast response statistics in a bottom-up way, we used stimuli that were simplified model images of natural scenes (‘dead leaves’, **Fig. 2A**), which have similar low-level structure as natural scenes (e.g. 1/f power spectra) but are devoid of semantic content. These images are created by filling a frame with objects - much like fallen leaves can fill a forest floor – and are used in computer vision to study, for example, how the appearance and the distribution of these objects influences the low-level structure of natural scenes [28]. By manipulating properties of the objects in a controlled manner, we created distinct image categories, and then tested whether differences between these categories in contrast statistics matched with behaviorally perceived similarity by letting human observers perform a same-different categorization task on all combinations of image categories.

Specifically, we used the space formed by the two Weibull parameters to compute geometric distances between images in contrast statistics, and used these distances as quantitative predictors of dissimilarity [29]–[31]. We thus tested whether these parameters can predict the extent to which image categories induced dissimilar single-image EEG responses (experiment 1) and whether they match with perceptual categorization at the behavioral level (experiment 2). We predicted that images with very different Weibull statistics would appear less similar, i.e. be less often confused than images from categories with similar statistics.

By using controlled images that we quantified using a model originally derived from contrast responses to natural images, we aim to build a bridge between findings obtained with systematic manipulation of artificial stimuli and those obtained with more data-driven natural scene studies. For purpose of comparison, and to better understand which statistical information is captured by the Weibull parameters, we also tested two other global contrast statistics (**Fig. 2C**). Following [32] we calculated the intercept and slope of the average power spectrum to parameterize spatial frequency information, a commonly used measure of low-level information in scene perception. In addition, we followed [33] to derive the skewness and kurtosis of the contrast distribution for a range of spatial scales: these higher-order properties of distributions have previously been suggested (e.g. [34], [35] to reflect low-level differences between images that are relevant for perceptual processing.

We find that Weibull statistics explain substantial variance in evoked response amplitude to the dead leaves images, predicting clustering-by-category of occipital ERP patterns within 100 ms of visual processing. In addition, they correlate with human categorization behavior: specific confusions were made between categories with similar Weibull statistics. By comparison, Fourier power spectra and skewness and kurtosis can be used for accurate classification of image category, but fail to predict neural clustering and behavioral categorization. These convergent results provide evidence for relevance of pooled contrast response statistics in rapid neural computation of perceptual similarity.

## Materials and Methods

### Ethics statement

The experiments reported here were approved by the Ethical Committee of the Psychology Department at the University of Amsterdam; all participants gave written informed consent prior to participation and were rewarded with study credits or financial compensation (7 euro/hour).

### Stimuli

Gray-scale dead leaves images (512×512 pixels, bit depth 24) were generated using Matlab. Images contained randomly placed disks that were manipulated along 4 dimensions (opacity, depth, size and distribution) to create 16 categories. Disks were either opaque or transparent; intensity at the outer edges of the disk was either constant (leading to a 2D appearance) or decaying (3D appearance), and disk size was determined by drawing randomly from a range of small, medium or large diameters (exact settings as in [28]. Twelve categories were created by systematically varying these properties of power-law distributed disks. Four more categories were created using medium-diameter, exponentially distributed disks that could be 2D or 3D and opaque or transparent. For each category, 16 images were created using these category-specific settings: the random placement and use of ranges of diameter sizes ensured that each of these 16 images was unique. This procedure thus resulted in a total of unique 256 images, divided into 16 distinct categories, which were used for experimentation (**Fig. 2A**).

#### Computation of contrast statistics

In the Weibull model, local contrast is computed at multiple spatial scales, after which a single optimal scale for each image location is selected. Subsequently, contrast responses are collected in a histogram that is summarized using a Weibull fit, yielding two statistical parameters: beta and gamma (**Fig. 2B**). For comparison with other contrast statistics, we computed spatial frequency statistics (using power spectra) and higher-order statistics (third moments of the contrast distribution) for various receptive field sizes (**Fig. 2C**). The computational steps of each method are described in detail below.

#### Weibull contrast statistics

We computed image contrast according to the standard linear-nonlinear model. For the initial linear filtering step we used contrast filters modeled after well-known receptive fields of LGN-neurons [36]. As described in detail in [18] each location in the image was filtered using Gaussian second-order derivative filters spanning multiple octaves in spatial scale [37]. Based on our previous result [17] that the beta parameter was best approximated by a linear summation of X-like receptive field size output, whereas the gamma parameter correlated highest with Y-like receptive field size contrast, two separate spatial scale octave ranges were applied to derive the two summary parameters in the present multi-scale model. For the beta parameter, a bank of filters with 5 octave scales (4, 8, 16, 32, 64) standard deviation in pixels was used; for the gamma parameter, the filter bank consisted of octave scales 5, 10, 20, 40 and 80. The output of each filter was normalized with a Naka-Rushton function with 5 semi-saturation constants between 0.15 and 1.6 to cover the spectrum from linear to non-linear contrast gain control in LGN.

From the population of gain- and scale-specific filters, one filter response was selected for each location in the image using minimum reliable scale selection [38], a spatial scale control mechanism in which the smallest filter with output higher than what is expected to be noise for that specific filter is selected. The rationale behind this approach is that to arrive at a faithful scale-invariant contrast representation, the visual system selects spatial scale by minimizing receptive field size while simultaneously maximizing response reliability. Noise thresholds for each filter were determined in a separate set of stimuli (1800 natural images from the ImageNet natural scene database, [39]) and set to half a standard deviation of the average contrast present in that dataset for a given scale and gain. Applying the selected filter for each location in the image resulted in a 512×512 pixel contrast magnitude map, which was converted in a 256-bin histogram summarizing the contrast distribution of the image, to which the Weibull function was fitted by a maximum likelihood estimator (MLE). The Weibull function is given by:
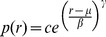
(1)where c is a normalization constant and μ, ß (beta) and γ (gamma) are the free parameters that represent the origin, scale and shape of the distribution, respectively. The value of the origin parameter μ is influenced by uneven illumination and generally close to zero for natural images. To achieve illumination invariance, this value was estimated and averaged out, leaving only the beta and gamma values as free parameters for each image.

#### Fourier power statistics

A two-parameter Fourier statistic was derived for each image by computing the intercept and slope of a line fitted to its power spectrum. We determined the power spectrum of the largest concentric square portion of the image (in this case, the entire image), excluding its outer edges to prevent edge artifacts. The cropped image was transformed into the frequency domain using the Fast Fourier Transform. Slope and intercept were estimated from the regression line fitted to the log-log representation of the power law-dependence:
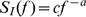
(2)The rotationally averaged power-law spectrum 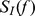 is defined as
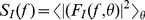
(3)where F*_I_ (f, θ)* is the Fourier transform spectrum of the input image *I*; *(f, θ)* are the cylindrical polar coordinates in Fourier space and 〈 〉*_θ_* denotes averaging over *θ*.

#### Contrast distribution statistics

Following [33], we used center-surround difference-of-Gaussian (DoG) filters to extract contrast values. Center receptive field sizes ranged between 2 and 4 pixels, and surround-to-center size ranged between 3 and 9, resulting in 21 different combinations of center size and surround-to-center ratio, referred to as receptive-field models. For each model, a scaling factor was used to set the integrated sensitivity of the surround to be 85% of that of the center. Per image, contrast responses were computed by convolving each pixel value with each of these 21 models separately. Responses were normalized using center-surround divisive normalization, where the difference in output of the center and surround is divided by their summed output. From the response distribution of responses across the image one skewness and one kurtosis value was derived for each image and for each receptive field model, resulting in 21 skewness and kurtosis values per image. Of these 21 values, results are reported for the skewness and kurtosis values that explained most EEG variance (center radius of 4 pixels with surround-center ratio 3); see next section (Experiment 1: EEG). This measure, computed exactly as reported in [33], has two important distinctions with the Weibull model, namely 1) the method does not incorporate scale selection; each receptive field model has one specific receptive field size that is used across the entire image and 2) only one parameter (skewness or kurtosis) is used to describe the response distribution that results from contrast filtering, compared to the separate scale (beta) and shape (gamma) parameters used in the Weibull model.

### Experiment 1: EEG

#### Experimental procedure

**Figure 3:**
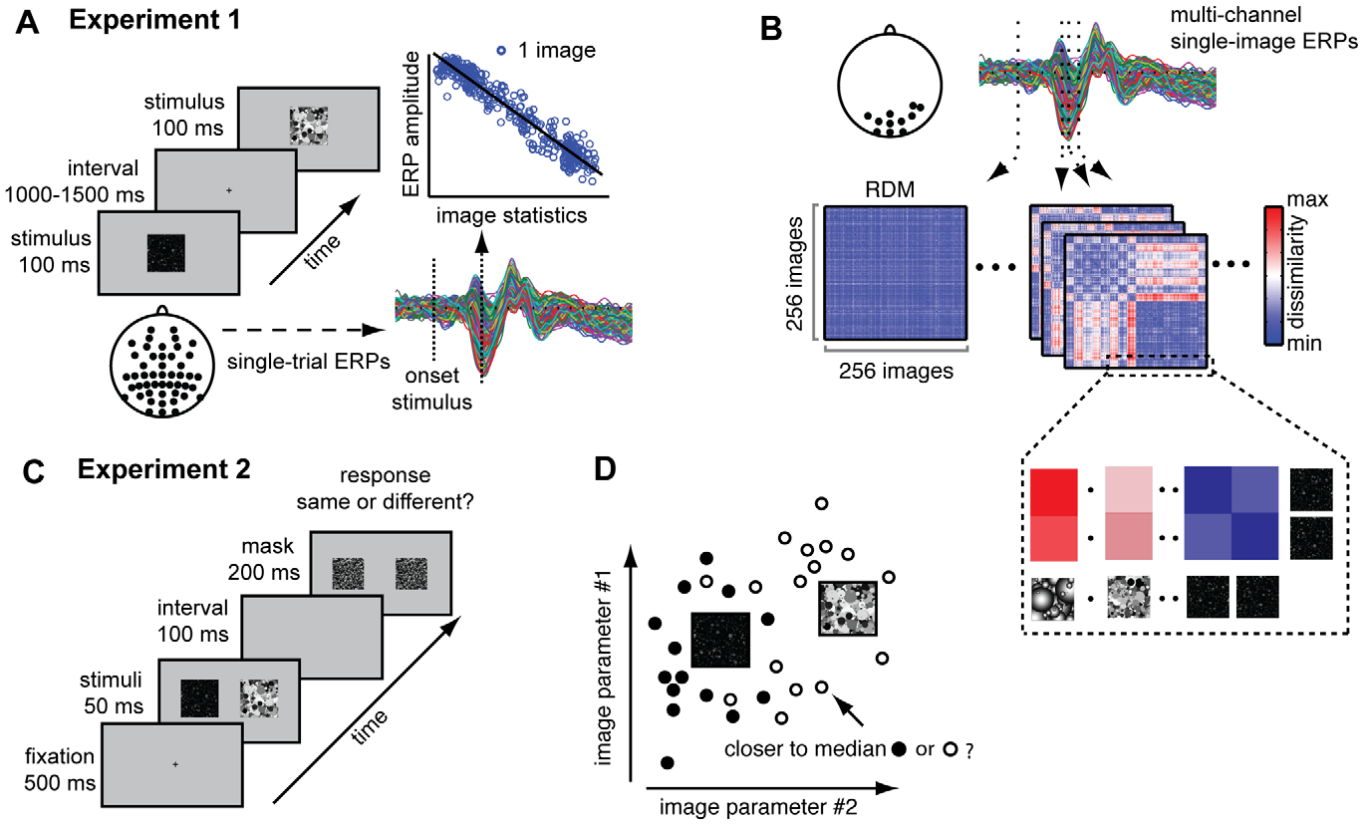
Methods and experimental design. (**A**), Experimental set-up of experiment 1 (EEG experiment). Subjects were presented with individual images of dead leaves while EEG was recorded. Single-image evoked responses (ERPs) were computed for each electrode, by averaging two repeated presentations of each individual image. Regression analyses of ERP amplitude on contrast statistics were performed at each time sample and electrode. (**B**), Representational dissimilarity matrices (RDMs) were computed at each sample of the ERP. A single RDM displays Euclidean distance (red = high, blue = low) between multiple-electrode patterns of ERP amplitude between all pairs of stimuli at a specific moment in time. The (cartoon) inset demonstrates how dissimilarities can cluster by category: all images from one category are in consecutive rows and can be ‘similarly dissimilar’ to other categories. (**C**), Experimental set-up of experiment 2 (behavioral experiment). On each trial, subjects were presented with a pair of stimuli for 50 ms, followed by a mask after an interval of 100 ms. Subjects were presented 8 times with all possible pairings of stimuli and were instructed to indicate whether stimuli were the same or different. (**D**), Cartoon example of leave-one-out classification based on contrast statistics. One stimulus is selected in turn, after which the median (thumbnail) of the remaining stimuli of its category is computed, as well as the median of other categories (here, just one). Classification accuracy reflects how many stimuli are closer to the median of other categories instead of its own category in terms of distance in image statistics.

Nineteen subjects took part in this experiment. The dead leaves images were presented on a 19 inch Ilyama monitor, whose resolution was set at 1024×768 pixels with a frame rate of 60 Hz. Subjects were seated 90 cm from the monitor such that stimuli subtended 11×11° of visual angle. During EEG acquisition, a single image was presented in the center of the screen on a grey background for 100 ms, on average every 1500 ms (range 1000–2000 ms; **Fig. 3A**). Each stimulus was presented twice, in two separate runs. Stimuli were presented intermixed with phase-scrambled versions of grayscale natural images; subjects were instructed to indicate which type of image they were shown. This instruction was intended to ensure that subjects attended to the stimuli: the required discrimination between the dead leaves and phase-scrambled natural images did not correspond to any distinction between the categories of dead leaves themselves. Examples of the two types of images were displayed prior to the experiment. Each run was subdivided in 8 blocks across which response mappings were counterbalanced. Stimuli were presented using the software package Presentation (www.neurobs.com).

#### EEG data acquisition

EEG Recordings were made with a Biosemi 64-channel Active Two EEG system (Biosemi Instrumentation BV, Amsterdam, NL, www.biosemi.com), with sintered Ag/AgCl electrodes at scalp positions including the standard 10-10 system along with intermediate positions and two additional occipital electrodes (I1 and I2), which replaced two frontal electrodes (F5 and F6). During recording, a CMS/DRL feedback loop was used as an active ground, followed by offline referencing to electrodes placed on the earlobes. The Biosemi hardware is completely DC-coupled, so no high-pass filter is applied during recording of the raw data. A Bessel low-pass filter was applied starting at 1/5^th^ of the sample rate. Eye movements were monitored with a horizontal electro-oculogram (hEOG) placed lateral to both eyes and a vertical electro-oculogram (vEOG) positioned above and below the left eye, aligned with the pupil location when the participants looked straight ahead. Data was sampled at 256 Hz.

#### EEG data preprocessing

The raw data was pre-processed using Brain Vision Analyzer by applying a high-pass filter at 0.1 Hz (12 dB/octave) and a low-pass filter at 30 Hz (24 dB/octave). Since this low-pass filter has a graded descent, it cannot be guaranteed that all high-frequency noise is removed; therefore, we additionally applied two notch filters at 50 (for line noise) and 60 Hz (for monitor noise). Deflections larger than 300 mV were automatically removed. Trials were segmented into epochs starting 100 ms before stimulus onset and ending at 500 ms after stimulus onset. These epochs were corrected for eye movements by removing the influence of ocular-generated EEG using a regression analysis based on the two horizontal and vertical EOG channels [40]. Baseline correction was performed based on the data between −100 ms and 0 ms relative to stimulus onset; artifacts were rejected using maximal allowed voltage steps of 50 µV, minimal and maximal allowed amplitudes of −75 and 75 µV and a lowest allowed activity of 0.50 µV (median rejection rate across subjects was 7%, with a range of 1%–38%). The resulting event-related potentials (ERPs) were converted to Current Source Density (CSD) responses [41]. This conversion results in a signal that is more localized in space, which has the advantage of more reliably reflecting activity of neural tissue underlying the recording electrode [42].

Trials in which the same individual image was presented were averaged over the two runs, resulting in a single event-related potential (ERP) for each image and each subject. To address the concern that regression results (see below) might be artificially high due to averaging of ERPs over repetitions, we also conducted all analyses using first-trial estimates only; these are reported in **Fig. S4** and **S5**; the results were very similar to those obtained with repetition-averaged ERPs.

#### Regression on single-image ERPs

To test whether differences between evoked neural responses could be predicted by differences in contrast statistics between images, we conducted regression analyses on the single-image ERPs (**Fig. 3A**). The preprocessed ERPs were read into Matlab, where we conducted linear regression analyses of ERP amplitude on image parameters using the Statistics Toolbox. For each subject, each channel and each time-point, two image parameters (Weibull parameters; Fourier parameters; skewness/kurtosis) were entered together as linear regressors on ERP amplitude. This analysis results in a measure of model fit (r^2^) over time (each sample of the ERP) and space (each electrode) for each individual subject.

To compare the results between different sets of statistics directly (within each subject), we used the Akaike information criterion (AIC, [43] which measures the information contained in each set of predictors. In this procedure, we transformed the residual sum of squares (RSS) of the regression analysis based on each set of statistics into AIC-values using AIC = n*log(RSS/n)+2k where n = number of images and k is the number of predictors. AIC can be used for model selection given a set of candidate models of the same data, where the preferred model has minimum AIC-value [44].

To test whether the various image parameters explained any unique variance, we ran an additional regression analysis using a full model in which all three sets of image statistics were entered simultaneously (resulting in a 6 parameter model). We compared the results obtained with the full model with models for which, in turn, each parameter was left out; by subtracting the r^2^ values of each of these partial models from the full model, we quantified unique variance explained by individual predictors.

To correct for multiple comparisons, the p-values associated with the regression results were FDR-corrected at α = 0.05, unless stated otherwise.

#### Representational similarity analysis

To examine how variance between individual visual stimuli arises over time and space, we computed representational dissimilarity matrices (RDMs; [25]) based on spatial patterns of evoked ERP amplitude. In this type of analysis, dissimilarity between patterns of activity evoked by individual images (measured as 1-correlation or Euclidean distance) is determined across multiple recording sites simultaneously (e.g., voxels in fMRI, [45]). Here, we computed RDMs based on ERP amplitude at each time-point, using the spatial pattern of evoked activity across multiple electrode sites; we did this for each subject separately. Only electrodes showing substantial variance across the entire stimulus- and dataset were included (**Fig. S1**); these were I1, I2, Iz, O1, O2, Oz, POz, PO7, PO8, P6 and P8. Based on this multi-electrode data, we computed (per subject and time-point) for all pairs of images the Euclidean distance between their evoked ERP amplitude patterns. As a result, we obtained RDMs containing 256×256 ‘dissimilarity’ values at each time-point of the ERP (**Fig. 3B**). Within one RDM, each cell reflects similarity in ERP amplitude patterns of the corresponding two images indicated by the row- and column number. We used Euclidean distance to quantify dissimilarity rather than the 1–correlation measure recommended for fMRI data [45] because it corresponds more closely to the distance measure taken for the contrast statistics matrices (see below).

#### Comparison with distance matrices based on contrast statistics

To examine whether the dissimilarities between ERP patterns evoked by individual images could be predicted based on differences in contrast statistics, we computed pair-wise dissimilarity matrices based on the three sets of parameter values (Weibull statistics; Fourier statistics; distribution statistics). We computed the sum of the absolute differences between the (normalized) parameter values of each pair of images (reflecting distance in the parameter space formed by the image parameters, **Fig. 1B**), resulting in one difference value between those two images. The matrices based on contrast statistics were compared with the RDMs based on the ERP data using a Mantel test for two-dimensional correlations [46], [47], denoted as r_m_. We computed these correlations for the average RDM across subjects as well as for single subjects RDMs. For the former, 95% confidence intervals for each correlation were assessed using a percentile bootstrap on the dissimilarity values [48] with number of bootstraps = 10.000 (∼40 * number of images).

### Experiment 2: Behavior

#### Behavioral data acquisition

Twelve participants took part in the behavioral experiment; none of them had participated in the EEG experiment. The dead leaves images were presented on a 19-inch Dell monitor with a resolution of 1280×1024 pixels and a frame rate of 60 Hz. On each trial, a fixation cross appeared at the center of the screen; after an interval of 500 ms, a pair of images was presented simultaneously for 50 ms, separated by a gap of 236 pixels (**Fig. 3C**). A mask followed after 100 ms, and stayed on screen for 200 ms. Participants were seated approximately 90 cm from the monitor; the stimulus display subtended 27×11° of visual angle. Subjects were instructed to indicate if the images were from the same or a different category by pressing one of two designated buttons on a keyboard (‘z’ and ‘m’) that were mapped to the left or the right hand. They completed four blocks of 256 trials each. In each block, the 256 trials were determined as follows: of the 16 images per category, 15 were paired with a randomly drawn image from another category (different-category comparisons); the 16th was paired with a randomly drawn image from the other 15 of its own category (same-category comparisons). Images were drawn without replacement, such that each image occurred only once in each block (with exception of the images that were selected for the same-category comparisons, which therefore occurred more often). Every possible different-category comparison thus occurred twice per block, and the ratio of different-category vs. same-category comparisons was 15∶1.

Before testing, subjects were informed that for most trials the stimuli were different, and that only some were the same, preventing them from adopting a balanced response (50-50) strategy. Also, subjects were shown a few example stimuli and performed 20 practice trials (none of which appeared in the main experiment) before starting the actual experiment. Masks were created by randomly placing four mini-blocks of 16×16 pixels from each of the 256 stimuli in a 512×512 frame. Unique masks were randomly assigned to each trial. The same mask was presented at the location of both stimuli. Stimuli were presented using the Matlab Psychophysics Toolbox [49], [50].

#### Behavioral data analysis

In total, each possible combination of the 16 categories was presented 8 times in 4 consecutive blocks. Trials at which the subject failed to respond (<1% for all subjects) within 1500 ms were discarded. Accuracy was determined by averaging across the four blocks. A mean confusion matrix was calculated by averaging accuracies across subjects separately for each specific combination of categories; we also calculated these matrices for each individual subject. We correlated both the mean confusion matrix and the individual matrices with classification accuracy based on contrast statistics (see below) using the Mantel test, resulting in one ‘mean’ and 12 individual correlation values. For these comparisons, the same-category comparisons were excluded (the Mantel test requires zero-values on the diagonal); they are included in the overall accuracy scores. Confidence intervals were determined using a percentile bootstrap (with number of bootstraps = 1000), which results in a 95% confidence interval along with the correlation.

#### Classification analysis on contrast statistics

To compare the behavioral performance with distance in contrast statistics, we performed leave-one-out classification analyses based on the parameter values of each set of contrast statistics (Weibull statistics; Fourier statistics; skewness/kurtosis). We used a simple algorithm that determines a single measure of classification accuracy based on the amount of overlap between different categories in parameter values. This involved the following steps: First, the median parameter values of each category were calculated. In turn, one of the 256 stimuli was selected, after which a temporary median of other 15 stimuli of its own category was determined. Next, the difference between its parameter values (beta and gamma for Weibull statistics; intercept and slope for Fourier statistics; skewness and kurtosis for distribution statistics) and the temporary median of its own category was calculated, as well as the difference with the median of all other categories. If the difference with its own category was less than the difference with any other category, this stimulus was counted as a ‘hit’, otherwise it was assigned a ‘miss’ (a cartoon example is shown in **Fig. 3D**). Classification accuracy was determined by counting the percentage of hits out of all comparisons. To determine significance, binomial density probabilities across all combinations in the dataset were calculated (the likelihood of a hit occurring rather than a miss) based on which an FDR-threshold was established that was used to correct the pair-wise classification accuracy values for multiple comparisons. Using the mean values for each category rather than the median to determine distances between images between yielded very similar results as those reported here.

## Results

### Contrast statistics

**Figure 4:**
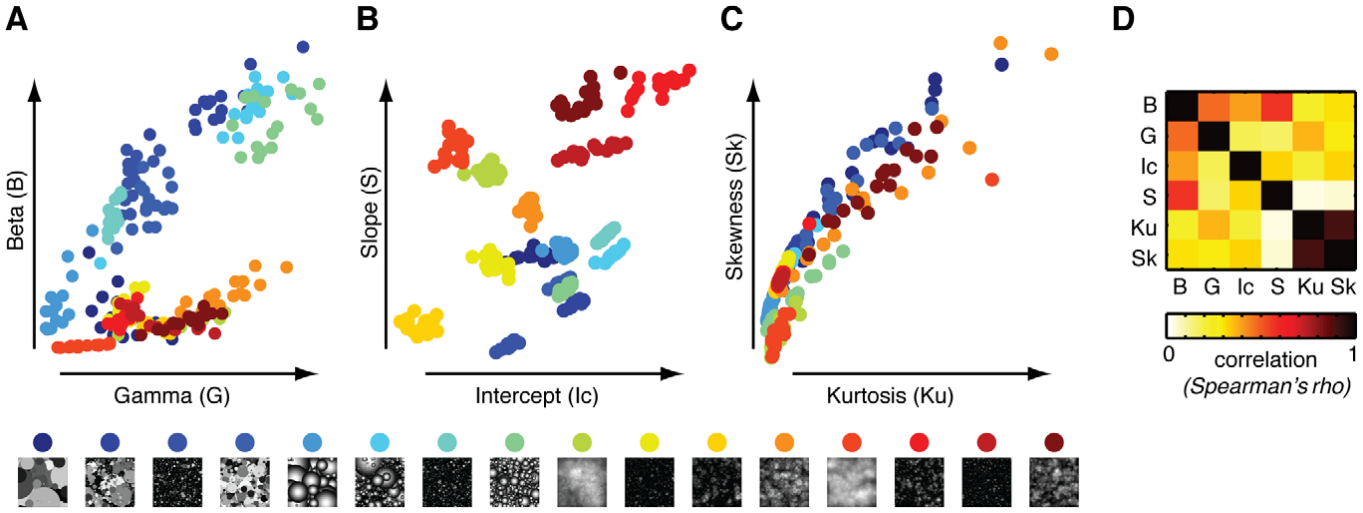
Stimuli set out against their respective contrast statistics. Each data-point reflects parameter values for a single image, color-coded by category. Individual images are displayed against their (**A**), Weibull parameters beta and gamma, (**B**), Fourier parameters intercept and (increasing negative) slope and (**C**), distribution properties skewness and kurtosis. In all cases, clustering by category based on parameter values is evident. (**D**), Non-parametric correlations between the six image parameters: Beta (B), Gamma (G), Fourier Intercept (Ic), Fourier Slope (S), Skewness (Sk) and Kurtosis (Ku).

If we set out all 256 dead leaves images against the three sets of image statistics (Weibull parameters, Fourier parameters and skewness/kurtosis), stimuli cluster by category in all cases, with Fourier parameters leading to the most separable clusters (**Fig. 4A–C**). There were considerable correlations between the various parameters (**Fig. 4D**; individual correlations plots in **Fig. S2**). Skewness and kurtosis correlated highly (ρ = 0.91, p<0.0001), but other significant correlations are observed as well, for example between Fourier slope and the Weibull beta parameter (ρ = 0.57, p<0.0001) and also between the two Weibull parameters (ρ = 0.48, p<0.001). A correlation of similar magnitude was also observed [17] for natural scenes, supporting the notion that the dead leaves stimuli used here have similar low-level structure as natural stimuli.

Interestingly, however, the ‘similarity spaces’ formed by each set of parameters are quite different between the various models. If Weibull parameters determine the axes of the similarity space (**Fig. 4A**), highly cluttered images with many strong edges (e.g. 2D opaque stimuli with small disks) are located in the upper right corner (high gamma, high beta); images containing fewer edges (e.g. with larger disks) are found more on the left (low gamma); and most of the transparent stimuli, with weak edges, cluster together in the bottom of the space (low beta). For Fourier intercept and slope (**Fig. 4B**), transparent categories are highly separated across the space: however, most images with strong edges end up in a similar part of the space (low slope, high intercept). Based on either skewness or kurtosis (**Fig. 4C**), a few categories are distinct, but most tend to cluster together. These qualitative results suggest that all parameters are informative about clustering of image categories, but that they index different image properties.

Importantly, they give rise to different predictions about which categories should lead to similar evoked responses based on overlapping parameter values. We tested these predictions using the single-image ERP data.

### Experiment 1

#### Contrast statistics explain variance in occipital ERPs

**Figure 5:**
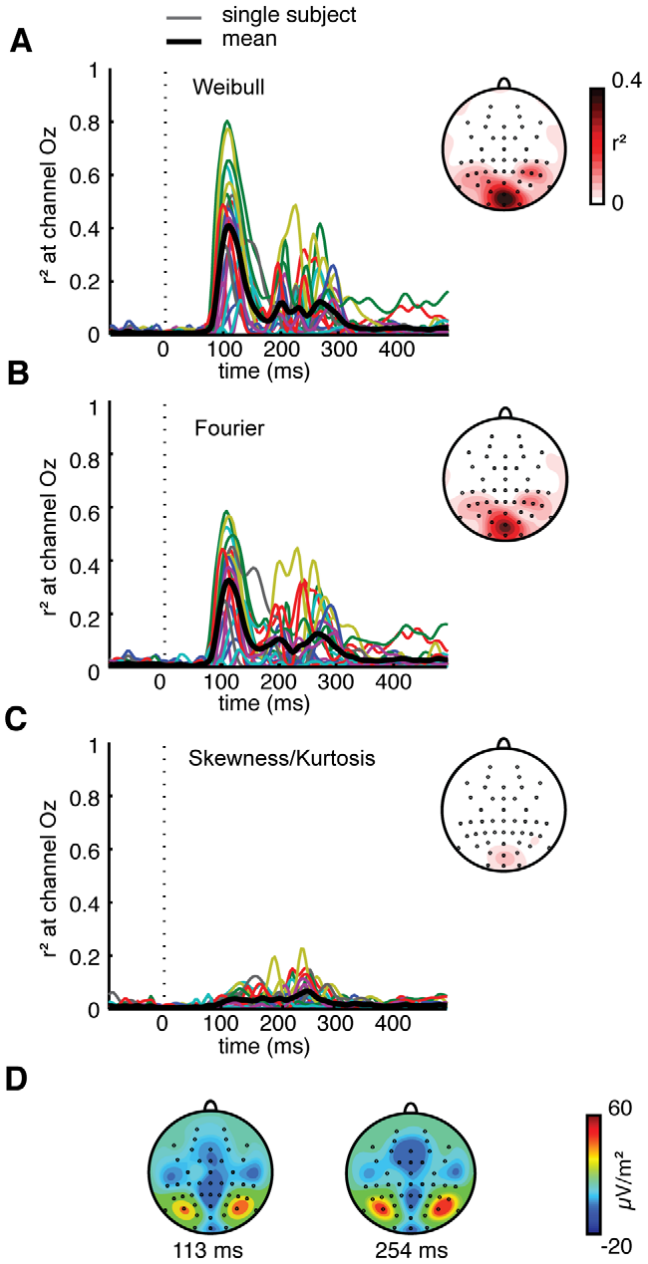
Regression analysis of EEG data: single subject results. Explained variance of ERP amplitude at channel Oz over time, for each individual subject (colored thin lines) and mean across subjects (black thick line), using as regressors either (**A**), Weibull parameters beta and gamma, (**B**), Fourier parameters intercept and slope and (**C**), skewness and kurtosis; single-trial results of these analyses can be found in **Fig. S4**. Insets display scalp plots of r^2^ values for all electrodes at the time of maximal explained variance averaged over subjects (113 ms for Weibull/Fourier, 254 ms for skewness/kurtosis. (**D**), Grand average ERP amplitude (averaged over all subjects and all images) for an early and a late time-point of peak explained variance displayed in **A–C**.

Regression of single-image ERP amplitude (per subject, electrode and time-point) on contrast statistics showed that Weibull statistics explain a substantial amount of variance between individual images. Highest values were found at occipital channel Oz, where explained variance for all subjects reached a maximum between 100 and 210 ms after stimulus onset; maximal values ranged between r^2^ = 0.12–0.80 (**Fig. 5A**) and were highly significant (all p<0.0001, FDR-corrected). For Fourier parameters (**Fig. 5B**), somewhat lower values were found (max r^2^ between 0.08–0.59, 100–210 ms; all p<0.0001). For skewness and kurtosis (**Fig. 5C**), explained variance was much lower and did not reach a consistent maximum during a specific time frame (max r^2^ between 0.02–0.23 at 78–421 ms; maximal values were significant for 11 out of 19 subjects).

If we average the explained variance across subjects for each electrode separately at the time-points of maximal explained variance (113 ms for Weibull and Fourier statistics, 254 ms for skewness/kurtosis), we see (insets **Fig. 5A–C**) that for all three sets of statistics, explained variance clusters around the midline occipital channels (Oz). Two weaker clusters were located near parietal electrodes, likely reflecting a dipole effect: both the early and late signals appear to originate from early visual areas (**Fig. 5D**).

These results demonstrate substantial differences in maximum explained variance between individual subjects. Inspection of the ERP recordings of each subject revealed a similarly large variability in subjects' signal-to-noise ratio (SNR, measured as the difference in ERP amplitude relative to pre-stimulus variability, reflecting the degree to which an evoked response is present). Indeed, the rank correlation between SNR and maximal explained variance by Weibull statistics was ρ = 0.69, p<0.0014; see **Fig. S3**, which includes examples of subject-specific r^2^ values alongside their single-image ERPs). This suggests that the observed variability in maximum explained variance is related to these subject-specific differences in SNR, which are in turn likely due to individual differences in cortical folding, scalp conductivity and recording conditions.

In an alternative analysis performed on single-trial rather than single-image data (in which repeated presentations of the same stimulus were averaged, see Materials and Methods), we found slightly lower explained variance for all models (maximal r^2^ values: 0.71 for Weibull statistics, 0.52 for Fourier statistics, and 0.16 for skewness/kurtosis, respectively; see **Fig. S4**). Importantly, however, the relative differences between the sets of image parameters were fully consistent with those reported here.

Overall, the regression results show that Weibull contrast statistics, but also Fourier statistics, reliably predict activity evoked by individual dead leaves images at the individual subject level. To investigate differences between the contributions of the different image predictors, we ran several additional analyses that are described below.

#### Comparisons between different image parameters

**Figure 6:**
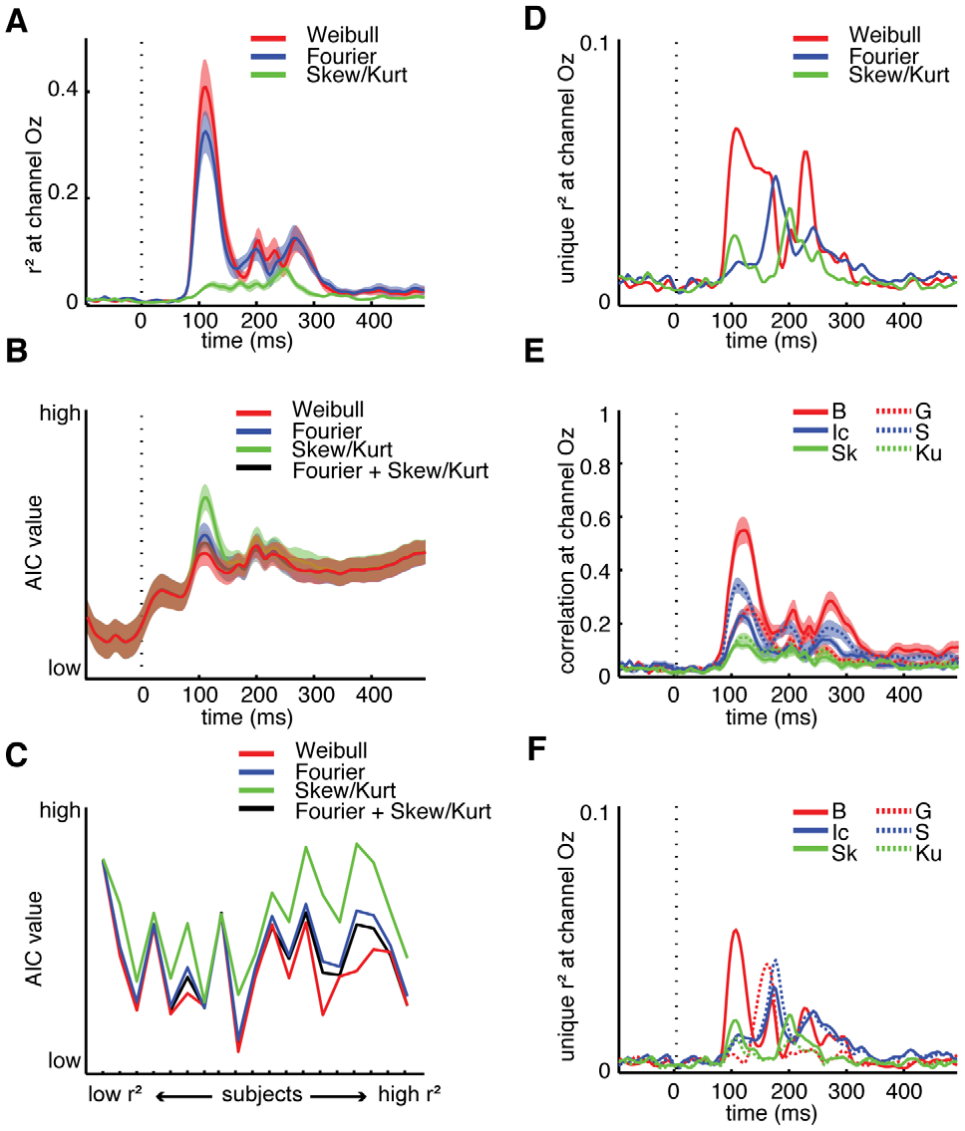
AIC (Akaike information criterion) and unique explained variance analyses at channel Oz. (**A**), Mean explained variance across single subjects for Weibull (red), Fourier (blue) and skewness/kurtosis (green), respectively; shaded areas indicate S.E.M. (**B**), Mean AIC-value across single subjects computed from the residuals of each of the three regression models, as well as an additional model (black) consisting of Fourier and skewness/kurtosis values combined, showing that Weibull parameters provide the best fit to the data (low AIC-value); shaded areas indicate S.E.M. (**C**), Single subject AIC-values for the models displayed in **B** at the time-point of maximal explained variance for Weibull and Fourier statistics (113 ms); subjects are sorted based on independently determined SNR ratio (reported in **Fig. S2**). (**D**), Unique explained variance by each set of contrast statistics. (**E**), Absolute, non-parametric correlations (Spearman's ρ) with ERP amplitude for the individual image parameters: Beta (B), Gamma (G), Fourier Intercept (Ic), Fourier Slope (S), distribution Skewness (Sk) and Kurtosis (Ku). Absolute values are plotted for convenience; shaded areas indicate S.E.M. (**F**), Unique explained variance by each individual parameter. Results for **A–E** based on single-trial rather than single-image data were highly similar (**Fig. S5**).

In order to compare differences in explained variance for Weibull statistics compared to the other statistics (**Fig. 6A**), we used Akaike's information criterion (AIC) to evaluate the relative ‘goodness of fit’ of each of the three sets of contrast statistics. AIC is computed from the residuals of regression analyses (see Materials and Methods) and can be used for model selection given a set of candidate models of the same data, where the preferred model has minimum AIC-value. If we compare the mean AIC-value across individual subjects of Weibull, Fourier and skewness/kurtosis parameters over time, we find that the model fits start to diverge around 100 ms, with Weibull statistics leading to the lowest values (**Fig. 6B**). It thus appears that Weibull parameters provide a better fit to the data than the other two sets of statistics. This could be related to the fact that the Weibull parameters characterize the histogram of contrast responses at a selected spatial scale, and may thus contain information reflected in both Fourier power spectra and higher-order properties of the contrast distribution. Therefore, we also computed AIC-values for intercept, slope, skewness and kurtosis combined into one regressor (**Fig. 6B**, black line); the obtained values from this regression analysis are however still higher than those obtained from the Weibull parameters (significant differences between 117–140 ms, all t(19)<−2.8, all p<0.01). At the time-point of (mean) maximal explained variance (113 ms), the ordering of the different models in terms of AIC-values is consistent over subjects (**Fig. 6C**): in all subjects, Weibull parameters lead to the best model fit, although differences are minimal for low SNR subjects. Interestingly, for subjects with high SNR, the distance between AIC-values for the Weibull model compared to the other contrast statistics appears to increase. These findings suggest that Weibull statistics capture additional variance relative to the other contrast statistics parameters considered here.

To demonstrate this in a different way, we computed the unique variance contributed by each set of contrast statistics (r^2^_unique_) by comparing partial models with a full model consisting of all 6 parameters (see Materials and Methods). Unique explained variance for each set of statistics was low (r^2^_unique_ for Weibull parameters reached a maximum of 0.07 at 109 ms; for Fourier, max r^2^_unique_ was 0.05 at 180 ms; for skewness/kurtosis, max r^2^_unique_ was 0.04 at 203 ms), but clearly highest for the Weibull parameters in an extended early time interval (∼100–180 ms; **Fig. 6D**). Given the substantial correlations between the various image parameters (reported in **Fig. 4D**), we also tested the contribution of each parameter individually. From the correlations of individual parameters with ERP amplitude (**Fig. 6E**), it can be readily seen that out of all parameters, the Weibull beta parameter correlates highest with the evoked activity in the early time-interval (max ρ = 0.57 at 121 ms, p<0.001 in 18 out of 19 subjects, FDR-corrected); it also has highest unique explained variance (r^2^_unique_ reaching a max of 0.05 at 109 ms, **Fig. 6F**), whereas the gamma parameter contributes unique variance somewhat later in time (max r^2^_unique_ was 0.04 at 164 ms), just before the Fourier parameters (a max of 0.03–0.04, around 175–180 ms).

Taken together, these additional analyses suggest that the differences in regression results between the various sets of contrast statistics reflect reliable and consistent differences in information about the stimulus carried by these statistics, with Weibull statistics resulting in the best fit to the differences observed in the neural data.

#### Clustering-by-category of ERPs is predicted by Weibull statistics

The regression results indicate that the Weibull parameters are predictive of ERP amplitude, but do not reveal whether any categorical differences between ERPs are reflected in these parameters. To address this, we constructed representational dissimilarity matrices (RDMs) based on EEG activity. In this analysis, we computed RDMs of ERP amplitude using multiple electrodes as input (see Materials and Methods) for each subject separately. This approach is akin to performing multi-voxel pattern analysis in fMRI and calculating the dissimilarity between these activity patterns, but now comparing ERP amplitude differences across electrodes instead of voxels. We computed one RDM for each time-point of the ERP and averaged RDMs over subjects.

**Figure 7:**
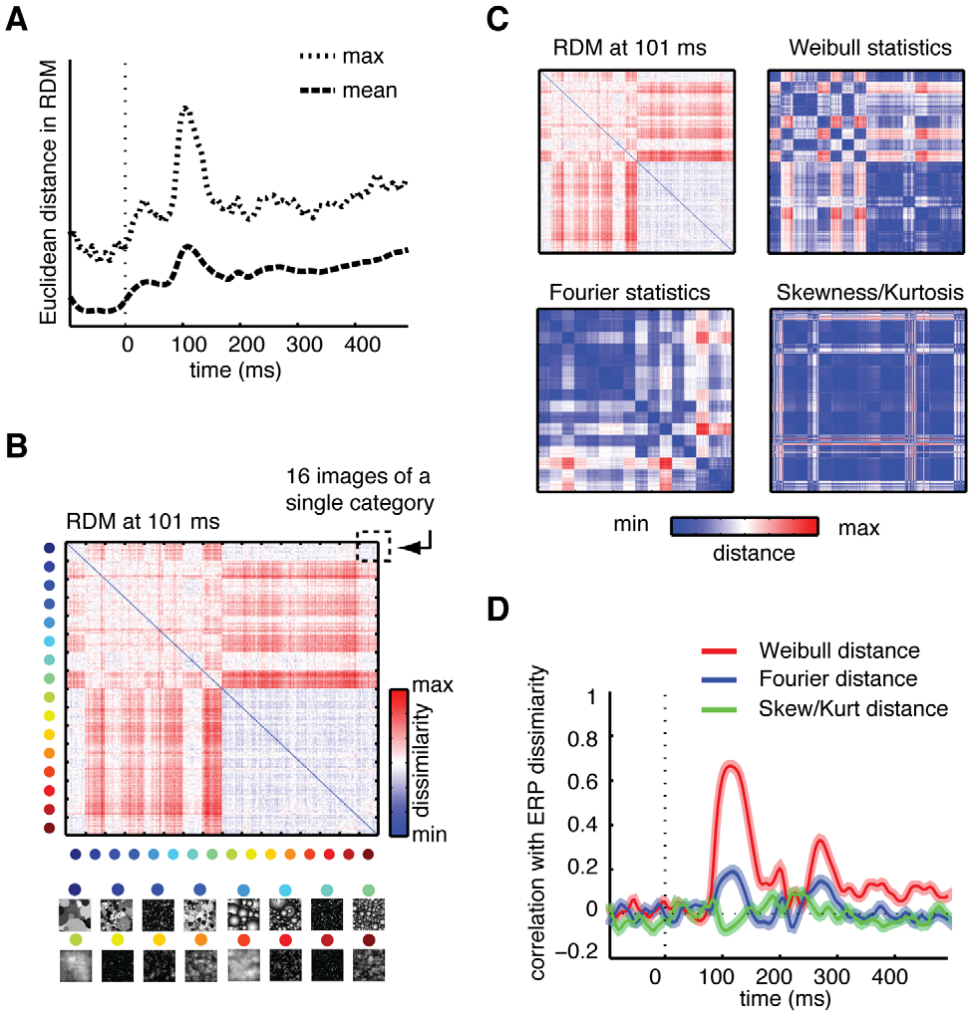
Results of RDM analysis. (**A**), Maximum and mean Euclidean distance for the subject-averaged RDM: for both measures, highest dissimilarity between images was found at 101 ms after stimulus-onset. (**B**), Mean RDM across subjects at the moment of maximal Euclidean distance. Each cell of the matrix reflects the dissimilarity (red = high, blue = low) between two individual images, whose category is indexed on the x- and y-axis. (**C**), Dissimilarity matrices based on difference in contrast statistics between individual images. Color values indicate the summed difference between two individual images in beta and gamma (Weibull statistics), intercept and slope (Fourier statistics), skewness and kurtosis (distribution statistics). (**D**), Correlation between the RDM and each of the three dissimilarity matrices at each time-point. Highest correlation is found for Weibull statistics at 109 ms. Shaded areas reflect 95% confidence intervals obtained from a percentile bootstrap on the dissimilarity values.

To demonstrate how these matrices can convey information about categorical properties of evoked responses, we selected the time-point at which maximal dissimilarities were found (**Fig. 7A**; 101 ms after stimulus-onset). In this subject-averaged RDM (**Fig. 7B**), we observe clustering by category: the matrix appears to consist of small blocks of 16×16 images that are minimally dissimilar amongst themselves (diagonal values), but that tend to differ from other categories (off-diagonal values). Moreover, differences between these blocks show that some categories are more dissimilar than others. Specifically, opaque categories (upper left quadrant) differ from one another and from transparent categories (lower left/upper right quadrant) whereas the transparent categories themselves tend to be minimally dissimilar (lower right quadrant).

Next, we tested to what extent these category-specific differences between images in the ERP were predicted by contrast statistics. We calculated 256×256 distance matrices for each set of image parameters, in which we subtracted the parameter values of each image from the values of each other image (**Fig. 7C**, see Materials and Methods). For example, for the first cell in the upper left corner of the Weibull statistics distance matrix, we summed the difference in beta and gamma values between image 1 and 2 (_bim1_−b_im2_+g_im1_−g_im2_), for the cell next to it between image 1 and 3, etc. For the other two sets of statistics, beta and gamma were replaced by intercept and slope or skewness and kurtosis.

By visual inspection alone, it is clear that distances between individual images in Weibull statistics are most similar to the ERP dissimilarities. Inter-matrix correlations (Mantel tests, [44]) reveal that at nearly all time-points there is a substantially higher correlation of the RDM of the ERP signal with the distance matrix based on Weibull, relative to the other two statistics (**Fig. 7D**). The highest correlations for Weibull and Fourier are found shortly after 100 ms (Weibull: r_m_ = 0.67, 109 ms; Fourier: r_m_ = 0.22, 113 ms) and are both significant after FDR-correction (p-values<0.001), whereas the correlation between the RDMs and the skewness/kurtosis distance matrix does not reach significance. We also correlated the distance matrices based on contrast statistics with the subject-specific RDMs, confirming this result to be consistent over subjects; see **Fig. S6**. RDMs at all ERP time-points are provided in **Video S1** in the form of a short movie clip.

These results show that differences between image categories in ERP amplitude map onto differences in underlying Weibull statistics of individual images. Throughout the ERP, this model of low-level visual responses provides a better prediction of differences between images in neural response patterns than the other image parameters considered here. Moreover, the highest correlation between differences in Weibull statistics and ERP amplitude is near the time-point of maximal dissimilarity, where clustering by category in the ERP is clearly present. This clustering corresponds to the categorical organization in Weibull parameter space (**Fig. 2A**), in which transparent categories were largely overlapping whereas stimuli with strong edges were more differentiated. In the next experiment, we asked whether this similarity space could not only predict early differences in ERP amplitude, but also behaviorally perceived similarity: do image categories with overlapping parameter values also look more alike?

### Experiment 2

#### Prediction of behavioral confusions

**Figure 8:**
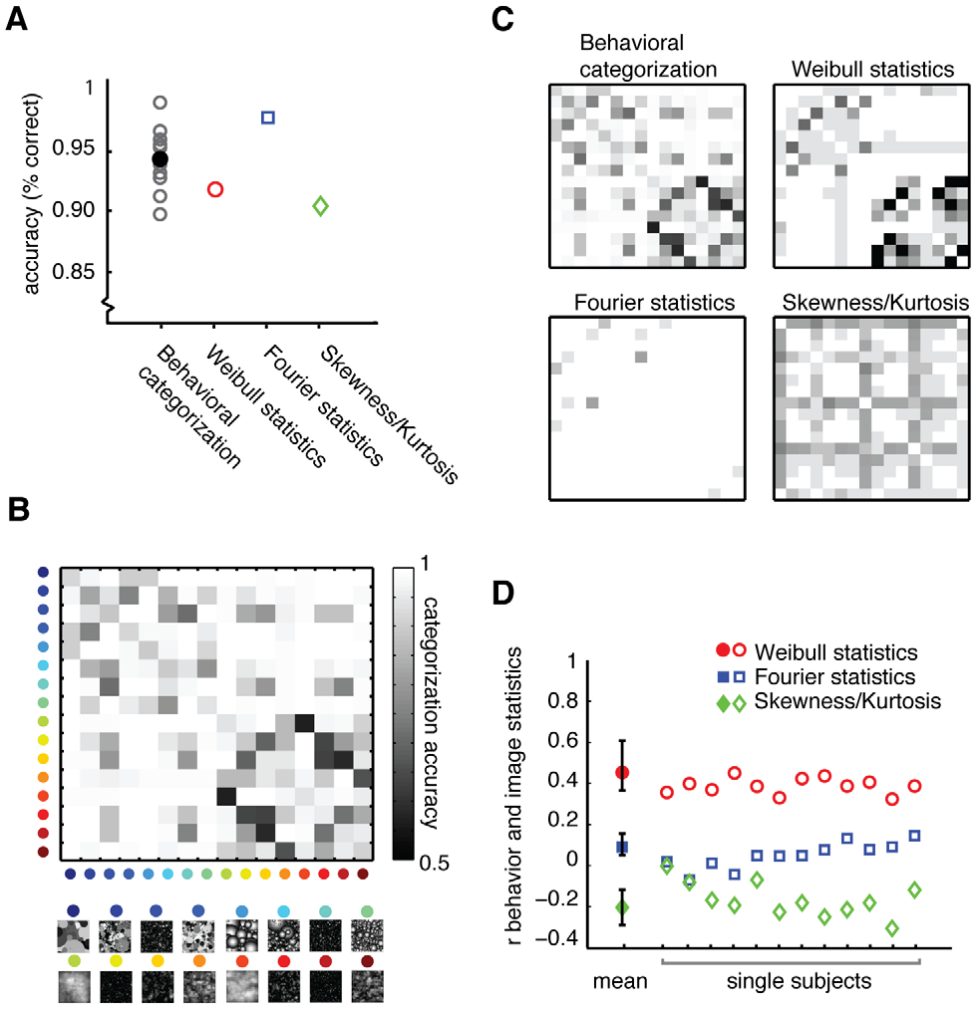
Behavioral results and comparison with classification. (**A**), Accuracy of behavioral categorization (open circles: single subjects, filled circle: mean) and of classification based on Weibull parameters, Fourier parameters or skewness and kurtosis. (**B**), Behavioral confusion matrix, displaying mean categorization accuracy for specific comparisons of categories. For each pair of categories the percentage of correct answers is displayed as a grayscale value. (**C**), Comparison of mean behavioral confusion matrix with classification results based on the three sets of contrast statistics. (**D**), Inter-matrix correlations of the classification errors for each set of statistics with the mean behavioral confusion matrix (left, mean) as well as those of individual participants (right, single subjects). For the mean correlation, error bars indicate 95% confidence intervals obtained using a percentile bootstrap on values within the mean confusion matrix.

Participants indicated for each possible combination of the 16 dead leaves categories whether these were the same or different category. Behavioral accuracy was high across all subjects (mean 93% correct, range 0.88–0.98), suggesting that subjects were well able to categorize these stimuli (**Fig. 8A**). To generate specific predictions about categorical similarity based on contrast statistics, we conducted classification analyses using the distance between images in each of the three similarity spaces, testing how often proximity in parameter values resulted in classification of an image to another category than its own (see Materials and Methods and **Fig. 3D**). Mean classification accuracy based on distance in contrast statistics was high for all three sets of contrast statistics, with highest accuracy for the Fourier parameters (99%), subsequently for the Weibull parameters (94%) and finally for skewness/kurtosis (93%). Despite these high accuracies, errors were made in both behavior and classification: to test whether these errors occurred for specific combinations of categories, we summarized the average number of errors for each specific combination of categories in confusion matrices.

From the mean behavioral confusion matrix (**Fig. 8B**), it is clear that subjects systematically confused certain categories more often than others. Specifically, transparent two- and three- dimensional images (dark squares in lower right quadrant) are more often confused than their opaque counterparts, although there were also some specific errors within opaque categories (upper left quadrant). Few errors were made between transparent and opaque categories. Although mean classification performance based on the Fourier parameters is highest, it is clear that the pattern of classification errors based on Weibull statistics most resembles the pattern of categorical confusions in behavior (**Fig. 8C**). As expected, the behavioral confusion matrix correlated significantly with classification errors made based on Weibull parameters (r_m_ = 0.46, p<0.001), whereas classification based on differences in Fourier parameters or skewness/kurtosis did not correlate with human performance (r_m_ = 0.07, p = 0.21 and r_m_ = −0.20, p = 0.03, respectively; although significant, a negative correlation indicates that classification errors are *opposed* to categorization errors made by human participants). Correlations of individual confusion matrices confirm this result across all subjects (**Fig. 8D**; range individual Weibull r_m_-values 0.33–0.46, all p<0.005, FDR-corrected).

These results show that perceived similarity of dead leaves image categories can be predicted based on differences in statistics of low-level contrast responses. Whereas mean classification accuracy for all image parameters was high, the different image parameters yielded different predictions about expected errors if categorization were to be based on these values. In the case of Fourier statistics, classification predicted that subjects would hardly confuse any categories at all, whereas skewness/kurtosis classification predicted that other categories would be confused with each other than those that subjects actually judged as similar. Only the Weibull parameters correlated with specific errors made by human subjects during rapid categorization.

This suggests that out of the three similarity spaces presented in **Fig. 4**, the arrangement of categories in Weibull space corresponds most closely to the actual perceptual similarity experienced by human subjects during a rapid categorization task.

## Discussion

Low-level contrast statistics, derived from pooling of early visual responses, can predict similarity of early visual evoked responses as well as perceptual similarity of model natural scene images. We show that Weibull statistics, derived from the output of contrast filters modeled after LGN receptive fields, correlate with perceived similarity of computationally defined dead leaves categories. These statistics explain a significant amount of variance in the early visual ERP signal and correlate with behavioral categorization performance. Based on differences in these statistics, we were able to predict specific dissimilarities in the neural signal as well as specific category confusions.

Interestingly, if we compare the results of experiment 1 and 2, we observe that subjects confused categories that were minimally dissimilar in ERP amplitude, which in turn were minimally different in Weibull statistics. Conversely, subjects accurately distinguished categories that were separable in their statistics, which was mirrored in high ERP dissimilarities. Also, correlations between Weibull statistics and neural responses were highest between 100 and 200 ms, well within the time frame that rapid categorization of natural images is thought to be constrained to [51].

This work extends recent findings that statistical variations in low-level properties are important for understanding categorical generalization over single images [13]. It has been demonstrated before that behavioral categorization can be predicted using computational modeling of low-level information: a neural network consisting of local filters that were first allowed to adapt to natural scene statistics could predict behavioral performance on an object categorization task [52], and a computational model based on texture statistics accurately predicted human natural scene categorization performance [53]. Here, we expand on these results by showing that a geometric ‘similarity space’ formed by low-level contrast statistics can predict a complex pattern of categorization confusions of model natural scene images.

### Implications for processing of real natural scenes

Whether low-level statistics are indeed actively exploited during scene or object categorization is a topic of considerable debate. Whereas some studies report that manipulation of low-level properties influences rapid categorization accuracy [54], [55] as well as early EEG responses [56], [57], other studies have shown that not all early visual activity is obliterated by equation of those properties [58]–[60] and, conversely, that early sensitivity to diagnostic information is revealed in stimuli that do not differ in low-level statistics [20], [61]. We find that, at least for our set of simplified models of natural scene images, early differences in ERPs are correlated with low-level contrast statistics that are themselves also directly predictive of perceptual similarity.

It is however likely that the degree to which low-level properties are relevant for processing of natural image categories is highly dependent on stimulus type and context, even within actual natural scene stimuli: for example, low-level information may influence rapid detection of faces to a larger extent than objects [22] and the effects of low-level statistics on animal detection may interact with scene category (man-made vs. natural) [62]. In addition, the present work is very different from these previous reports in that our experiments did not require formation of a high-level representation but only a same-different judgment. There are also notable differences between our ERP effects and those obtained with standardized object/scene categories: our maximum explained variance was found at around 100 ms, whereas those studies report sensitivity starting at 120 ms and onwards [63]–[66]. Maximal sensitivity of evoked activity to faces and objects is found at lateral-occipital and parietal electrodes (PO, e.g. [58]), whereas our correlations are clustered around occipital electrode Oz. This suggests that the dead leaves images may mostly engage mid-level areas of visual processing, such as those sensitive to textural information, e.g. V2 [24], [67]–[69]. Our results implicate that clustering of image similarities at this level of processing can, in principle, already predict perceptual similarity – in turn, these similarities can be derived from Weibull contrast statistics. Given that for natural scenes, the Weibull statistics explain similar amounts of variance in EEG activity as reported here, we can hypothesize that image similarities as predicted by Weibull statistics are also present in evoked activity to actual natural scenes.

### Information contained in contrast statistics

If Weibull statistics indeed approximate meaningful global information in natural images, which image features do they convey? By manipulating computational image categories in their perceptual appearance, we were able to get a better understanding of the information contained in the Weibull parameters. They appear to index the amount of clutter, i.e. are related to occlusion and object size. These properties may be relevant for natural scene categorization: a forest has a higher degree of clutter (high gamma) and lower mean edge strength (high beta) compared to a beach scene. An image containing a few strong edges (low beta) that are sparsely distributed (low gamma) has high probability of coinciding with a single salient object, for example a single bird against an empty sky, suggesting that these statistics may be relevant for object detection in natural scenes. Here, behavioral confusions (and corresponding dissimilarities in ERP signals) were found between stimuli without coherent edge information (transparent stimuli with either large or small disks), or that were highly cluttered (opaque stimuli with small disks) which were exactly the categories that overlapped in Weibull parameter values.

For comparison, we computed Fourier power spectra and higher-order properties of the contrast distribution (skewness and kurtosis), two sets of statistics that each index different sources of information in natural images: spatial frequency content and central moments of the contrast distribution, respectively. Deviations in the power spectra of natural images inform about variations in contrast across spatial scales: the slope and intercept parameters describe the ‘spectral signature’ of images [32] which is diagnostic of scene category [15]. Skewness and kurtosis were proposed to be relevant for texture perception [35], [70] which in turn can be important for feature detection [53], [71] and the presence of featureless regions of images [34], [72]. Our results confirm that both frequency content and central moments of the contrast distribution inform about image properties: both lead to accurate image classification. However, in the present study they did not predict neural and behavioral categorization patterns, suggesting that these statistics may not be plausible computations involved in visual processing of the dead leaves images.

Even though we used controlled, computationally defined image categories, it is still possible that an image property other that the contrast statistics tested here will provide a better prediction of the (neural and behavioral) data, for example one of the manipulations used to create the image categories (e.g., opacity). However, neither the observed clustering-by-category of ERPs in the RDM, nor the pattern of categorization errors in behavior mapped clearly onto one of the manipulations used to create the categories (e.g., opaque vs. transparent; as is visible in **Fig. 7B**, there are also differences *within* opaque and transparent categories, and this complex pattern of clustering is only predicted by Weibull statistics).

### Explaining the advantage of Weibull statistics

Why is the Weibull model better than widely used contrast statistics in predicting early neural and perceptual similarity? Although higher order moments of distributions can be diagnostic of textural differences, they may in practice be difficult for the visual system to represent [35]. In addition, it has been suggested that rather than amplitude spectra, phase information derived from the Fourier transform [73], [74], or the interaction between these two [75], [76] contains diagnostic scene information. The reason that higher-order statistics derived from the phase spectrum may contain perceptually relevant information [77] is that they carry edge information. In the Weibull model, contrasts, i.e. non-oriented edges, are explicitly computed (as the response of LGN-type neurons) and evaluated at multiple spatial scales. The model may thus be able to capture information contained both in power spectra (scale statistics) as well as central moments (distribution statistics). The Weibull parameters appear to reflect different aspects of low-level information: the beta parameter varies with the range of contrast strengths present in the image, reflecting overall *contrast energy*, whereas the gamma parameter varies with the degree of correlation between local contrast values, reflecting clutter or *spatial coherence*.

Obviously, the Weibull fit is still a mathematical construct. However, the two parameters can also be approximated in a more biologically plausible way: with our previous single-scale model [17], we demonstrated that simple summation of X- and Y-type LGN output corresponded strikingly well with the fitted Weibull parameters. Similarly, if the outputs of the multi-scale filter banks used here (reflecting the entire range of receptive field sizes of the LGN) are linearly summed, we again obtain values that correlate highly with the Weibull parameters obtained from the contrast histogram at minimal reliable scale (S. Ghebreab, H.S. Scholte, V.A.F. Lamme, A.W.M Smeulders, under review). This suggests that Weibull estimation can in fact be reduced to pooling of neuronal population responses by summation, which is a biologically realistic operation.

Why would summation of contrast responses of low-level neurons convey the same information as the Weibull parameters? This is likely a result of the structure of the world itself: distributions of contrast in natural images tend to range between power-law and Gaussian, which is the family of distributions that the Weibull function can capture [78]. It appears that this statistic simply provides a good characterization of the dynamic range of the low-level input to the visual cortex when viewing natural images. Since our brain developed in a natural world, early visual processing may take advantage of this regularity in estimating global properties to arrive at a first impression of scene content.

### Outlook

The present results extend our previous findings [17], [18] with natural images to other image types (computational categories) and to prediction of behavioral categorization. Interestingly, even though the subjects in experiment 1 (EEG) were not engaged in categorization of the dead leaves images, their results generalize to the behavioral categorization patterns that were found in experiment 2, suggesting that similarity of bottom-up responses measured in EEG - in a different person - can be predictive of the perceived similarity during categorization of these images. This observation is now restricted to computationally defined categories. An interesting question for future work is whether in construction of high-level categorical representations of natural stimuli - considered a computationally challenging task - the brain actively exploits the pattern of variability of the population response to low-level information, estimated from early receptive field output. Contrary to the classical view of the visual hierarchy (e.g., [79]) it has been proposed that a rapid, global percept of the input (gist) precedes a slow and detailed analysis of the scene [80]–[83]. Natural image statistics provide a pointer to information that could be relevant for such a global percept [84], [85]. However, the mechanism by which global information can be rapidly extracted from low-level properties is not directly evident from natural image statistics alone. As explained above, in our model, the statistics are derived from a biologically realistic substrate (the response of early visual contrast filters). We suggest that to build a realistic model of natural image categorization, it is essential to understand how statistics derived from very early, simple low-level responses can contribute to gist extraction.

In conclusion, our findings suggest that global information based on low-level contrast can be available very early in visual processing and that this information can be relevant for judgment of perceptual similarity of controlled image categories.

## Supporting Information

Figure S1 Selection of electrodes (Iz, I1, I2, Oz, O1, O2, POz, PO7, PO8, P6, P8) that were used as input to compute RDMs (dissimilarity matrices). Selection was based on standard deviation in ERP amplitude across the whole data set (all subjects and all images). Each line corresponds to a single electrode: only electrodes whose standard deviations crossed the dashed line were selected.
(TIF)

Figure S2 Correlations of individual image parameters Weibull beta (**A**) and gamma (**B**) with Fourier intercept, Fourier slope, skewness and kurtosis values.
(TIF)

Figure S3 Left: Correlation between subject-specific signal-to-noise ratio (SNR) and maximal explained variance (across all electrodes). SNR was computed by 1) per electrode, averaging the mean ERP amplitude across the 256 images over all post-stimulus time-points, 2) dividing the absolute value of this average by the standard deviation of all pre-stimulus time-points and 3) averaging the resulting SNR values over electrodes. The SNR-values thus reflect the degree to which stimulus-related ERP amplitude is present relative to baseline fluctuations. Right: two examples of evoked responses (CSD-transformed) for the 256 individual stimuli and corresponding explained variance values at channel Oz. Top: example of high SNR single-subject data; an ERP is clearly visible in individual trials; explained variance based on contrast statistics is high. Bottom: example of low SNR single-subject data; an evoked response is hardly discernable in the individual trials; explained variance based on contrast statistics is low. This result elegantly shows that if there is no evoked response present in the EEG signal, there is no stimulus-related variance to be explained by differences in contrast statistics.
(TIF)

Figure S4 Explained variance values at channel Oz as reported in **Fig. 5A–C**, but now computed based on non-averaged single-trial ERPs (compared to single-image ERPs that are averaged over repeats). As regressors, we used either (**A**), Weibull beta and gamma, (**B**), Fourier intercept and slope and (**C**), skewness and kurtosis. Colored thin lines: r^2^ values for individual subjects. Black thick line: mean r^2^ across subjects.
(TIF)

Figure S5 AIC and unique variance analyses at channel Oz as reported in **Fig. 6**, but now computed based on non-averaged single-trial ERPs (compared to single-image ERPs that are averaged over repeats). (**A**), Mean explained variance across subjects for Weibull (red), Fourier (blue) and skewness/kurtosis (green); shaded areas indicate S.E.M. (**B**), Mean AIC-value across single subjects computed from the residuals of each of the three regression models, as well as an additional model (black) consisting of Fourier and skewness/kurtosis values combined, shaded areas indicate S.E.M. (**C**), Single subject AIC-values at the time-point of maximal explained variance for Weibull and Fourier statistics (113 ms); subjects are sorted based on SNR ratio (reported in **Fig. S2**). (**D**), Unique explained variance by each set of contrast statistics. (**E**), Absolute, non-parametric correlations (Spearman's ρ) with ERP amplitude for the individual image parameters: Beta (B), Gamma (G), Fourier Intercept (Ic), Fourier Slope (S), distribution Skewness (Sk) and Kurtosis (Ku). Absolute values are plotted for convenience; shaded areas indicate S.E.M. (**F**), Unique explained variance by each individual image parameter.
(TIF)

Figure S6 Single-subject correlations of dissimilarity matrices (RDMs) of ERPs with distance matrices based on the three sets of contrast statistics: (**A**), Weibull parameters, (**B**), Fourier parameters and (**C**), skewness and kurtosis.
(TIF)

Video S1 Representational dissimilarity matrices at each sample in time of the ERP, starting 50 ms before until 350 ms after stimulus-onset. Dissimilarity between stimuli is measured as Euclidean distance (red = maximal, blue = minimal values in entire data set) between ERP patterns across occipital electrodes (see Materials and Methods). Categories are labeled on the x- and y-axis; each cell of the matrix indexes the dissimilarity between two individual stimuli. Differences between images suddenly emerge around 90 ms after stimulus-onset and disappear again about 60 ms later. These differences cluster in 16×16 blocks, suggesting that categorical information is present in this time period. Later in time, weaker differences arise, but not as large as before, suggesting that category-specific dissimilarities between stimuli are evoked early in time.
(MPG)
