# Supplementary figures and images for: Spatially Pooled Contrast Responses Predict Neural and Perceptual Similarity of Naturalistic Image Categories
Source: PLoS Comput Biol. 2012 Oct 18;8(10):e1002726. doi: 10.1371/journal.pcbi.1002726 (PMC3475684; doi:10.1371/journal.pcbi.1002726)

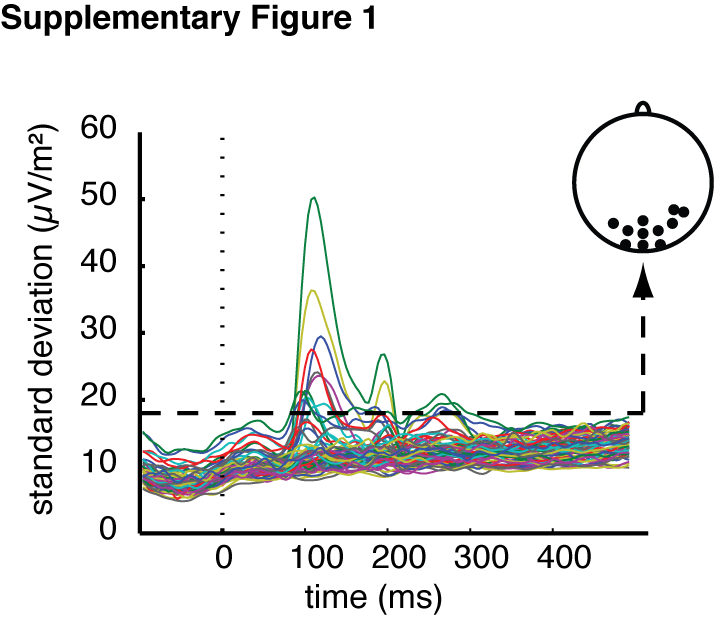

Supplement: Figure S1 — Selection of electrodes (Iz, I1, I2, Oz, O1, O2, POz, PO7, PO8, P6, P8) that were used as input to compute RDMs (dissimilarity matrices). Selection was based on standard deviation in ERP amplitude across the whole data set (all subjects and all images). Each line corresponds to a single electrode: only electrodes whose standard deviations crossed the dashed line were selected. (TIF) [file pcbi.1002726.s001.tif]

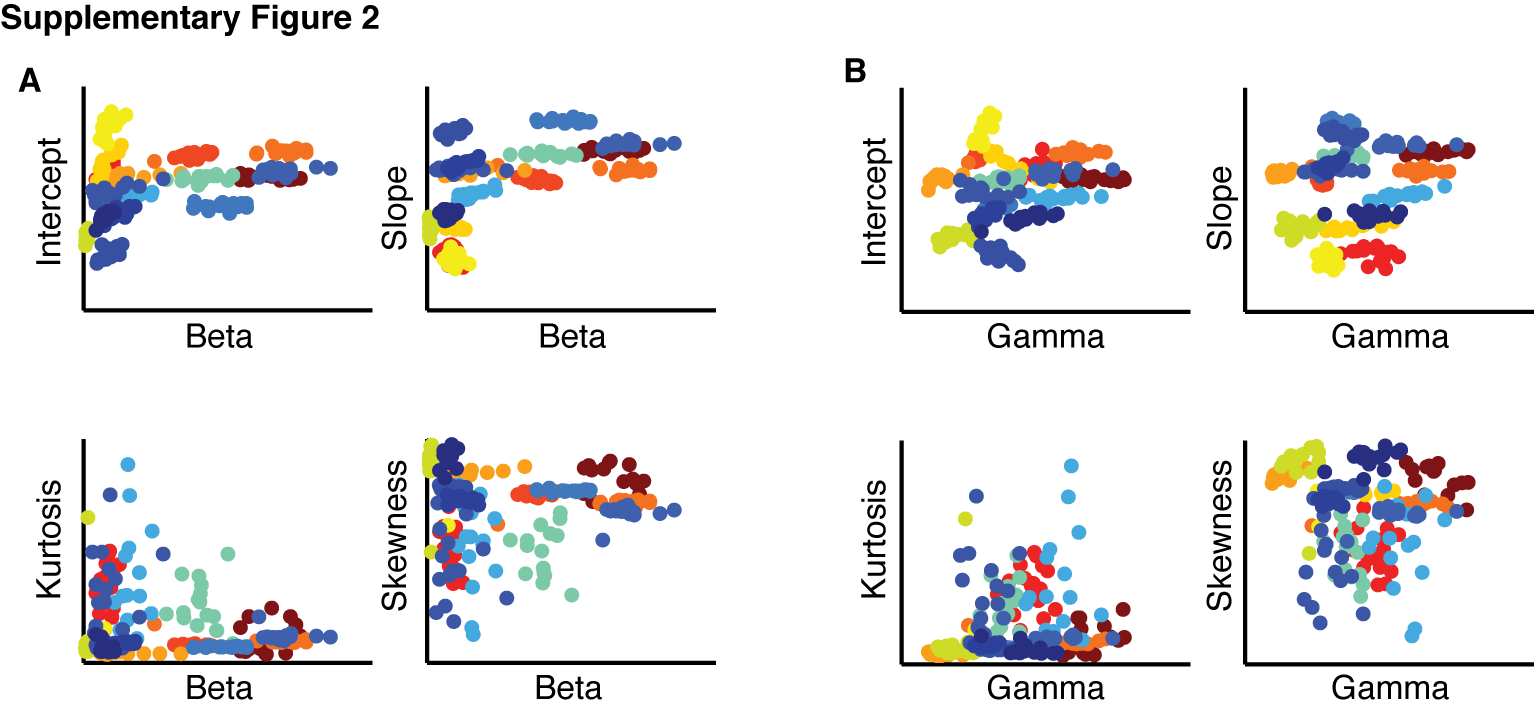

Supplement: Figure S2 — Correlations of individual image parameters Weibull beta (A) and gamma (B) with Fourier intercept, Fourier slope, skewness and kurtosis values. (TIF) [file pcbi.1002726.s002.tif]

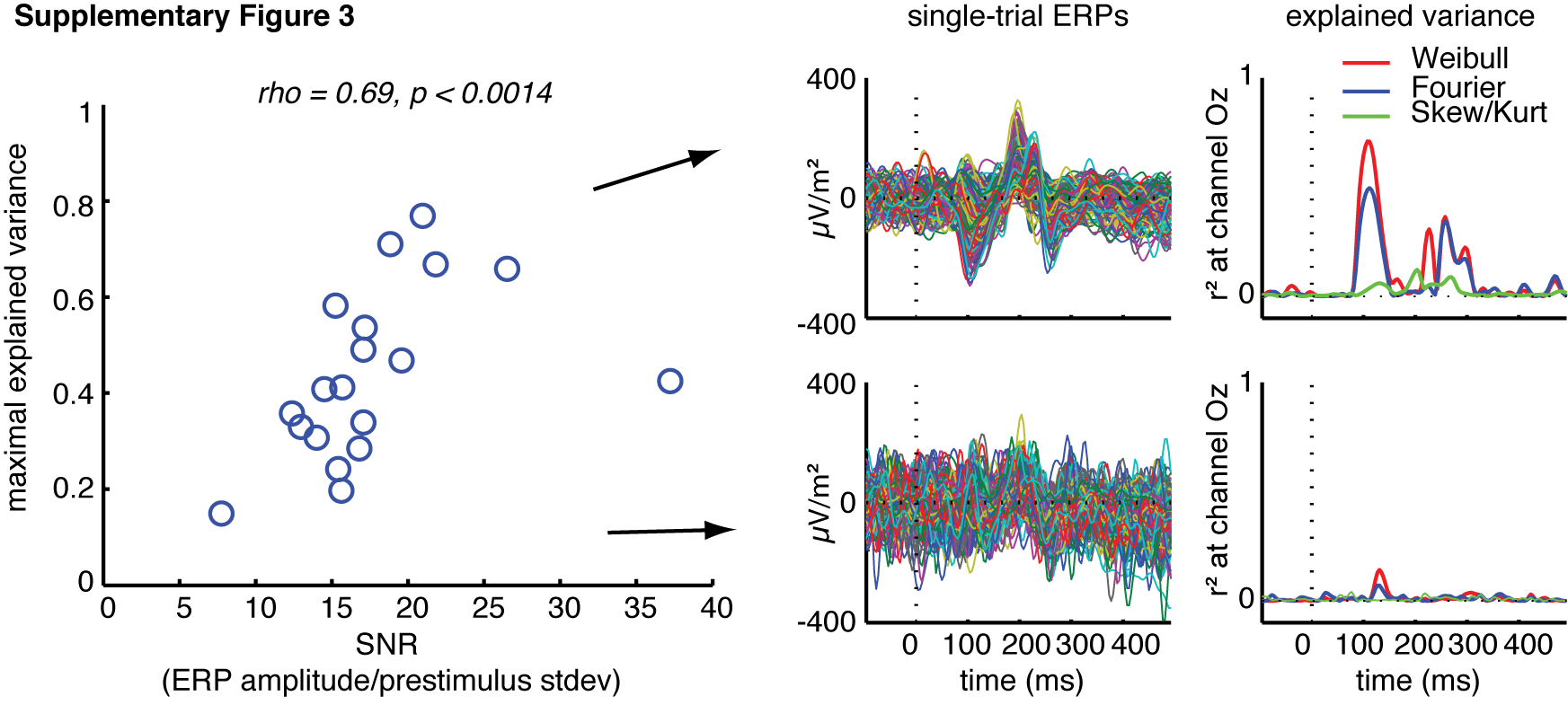

Supplement: Figure S3 — Left: Correlation between subject-specific signal-to-noise ratio (SNR) and maximal explained variance (across all electrodes). SNR was computed by 1) per electrode, averaging the mean ERP amplitude across the 256 images over all post-stimulus time-points, 2) dividing the absolute value of this average by the standard deviation of all pre-stimulus time-points and 3) averaging the resulting SNR values over electrodes. The SNR-values thus reflect the degree to which stimulus-related ERP amplitude is present relative to baseline fluctuations. Right: two examples of evoked responses (CSD-transformed) for the 256 individual stimuli and corresponding explained variance values at channel Oz. Top: example of high SNR single-subject data; an ERP is clearly visible in individual trials; explained variance based on contrast statistics is high. Bottom: example of low SNR single-subject data; an evoked response is hardly discernable in the individual trials; explained variance based on contrast statistics is low. This result elegantly shows that if there is no evoked response present in the EEG signal, there is no stimulus-related variance to be explained by differences in contrast statistics. (TIF) [file pcbi.1002726.s003.tif]

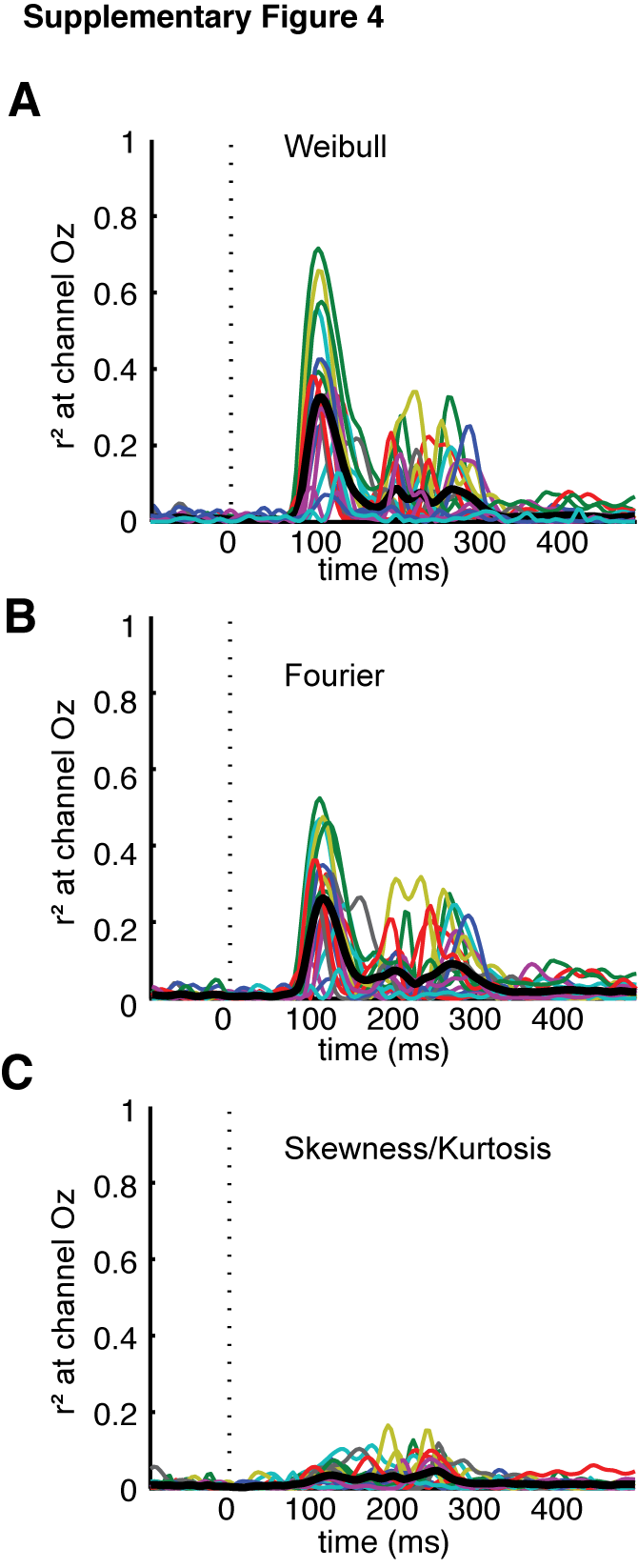

Supplement: Figure S4 — Explained variance values at channel Oz as reported in Fig. 5A–C , but now computed based on non-averaged single-trial ERPs (compared to single-image ERPs that are averaged over repeats). As regressors, we used either (A), Weibull beta and gamma, (B), Fourier intercept and slope and (C), skewness and kurtosis. Colored thin lines: r2 values for individual subjects. Black thick line: mean r2 across subjects. (TIF) [file pcbi.1002726.s004.tif]

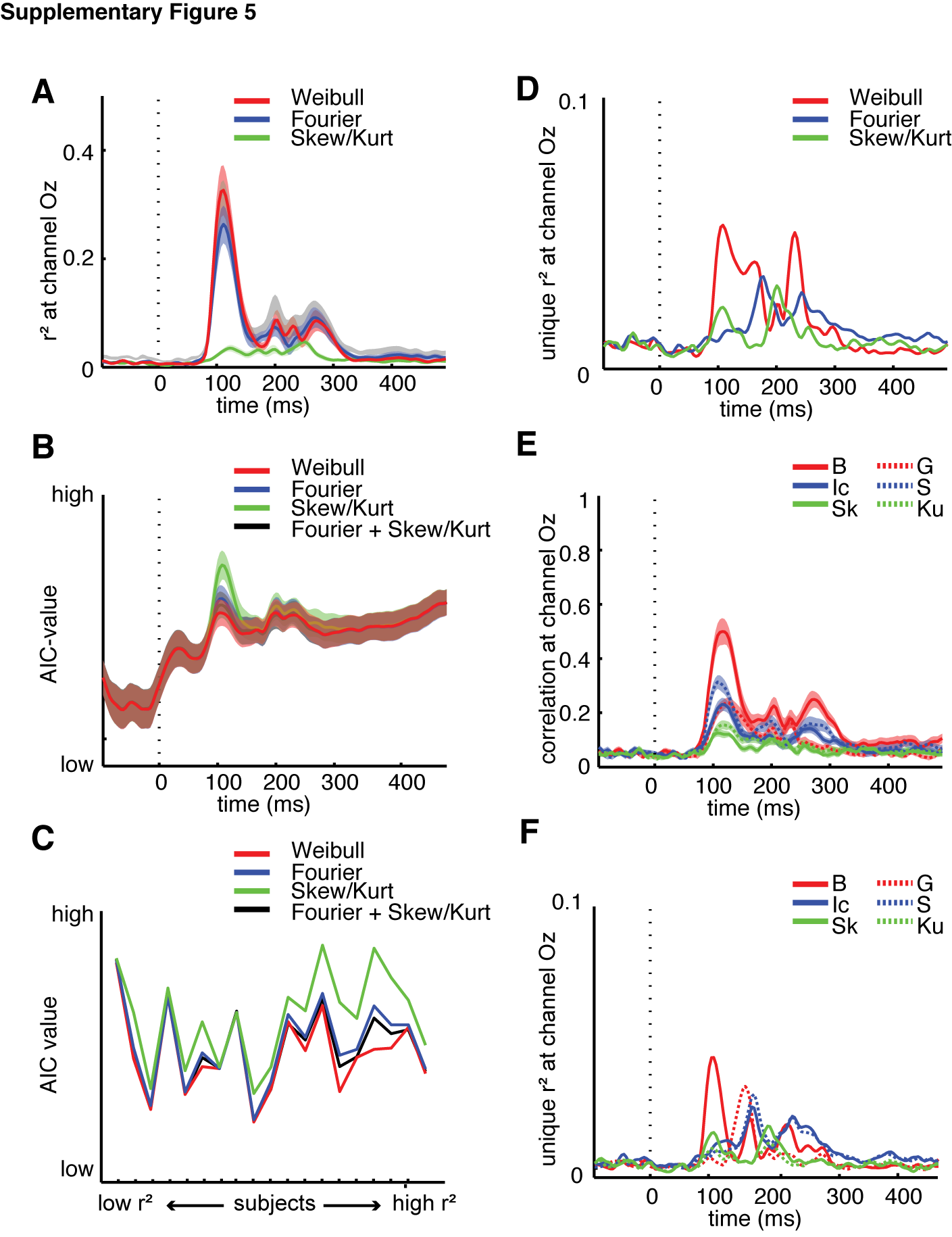

Supplement: Figure S5 — AIC and unique variance analyses at channel Oz as reported in Fig. 6 , but now computed based on non-averaged single-trial ERPs (compared to single-image ERPs that are averaged over repeats). (A), Mean explained variance across subjects for Weibull (red), Fourier (blue) and skewness/kurtosis (green); shaded areas indicate S.E.M. (B), Mean AIC-value across single subjects computed from the residuals of each of the three regression models, as well as an additional model (black) consisting of Fourier and skewness/kurtosis values combined, shaded areas indicate S.E.M. (C), Single subject AIC-values at the time-point of maximal explained variance for Weibull and Fourier statistics (113 ms); subjects are sorted based on SNR ratio (reported in Fig. S2). (D), Unique explained variance by each set of contrast statistics. (E), Absolute, non-parametric correlations (Spearman's ρ) with ERP amplitude for the individual image parameters: Beta (B), Gamma (G), Fourier Intercept (Ic), Fourier Slope (S), distribution Skewness (Sk) and Kurtosis (Ku). Absolute values are plotted for convenience; shaded areas indicate S.E.M. (F), Unique explained variance by each individual image parameter. (TIF) [file pcbi.1002726.s005.tif]

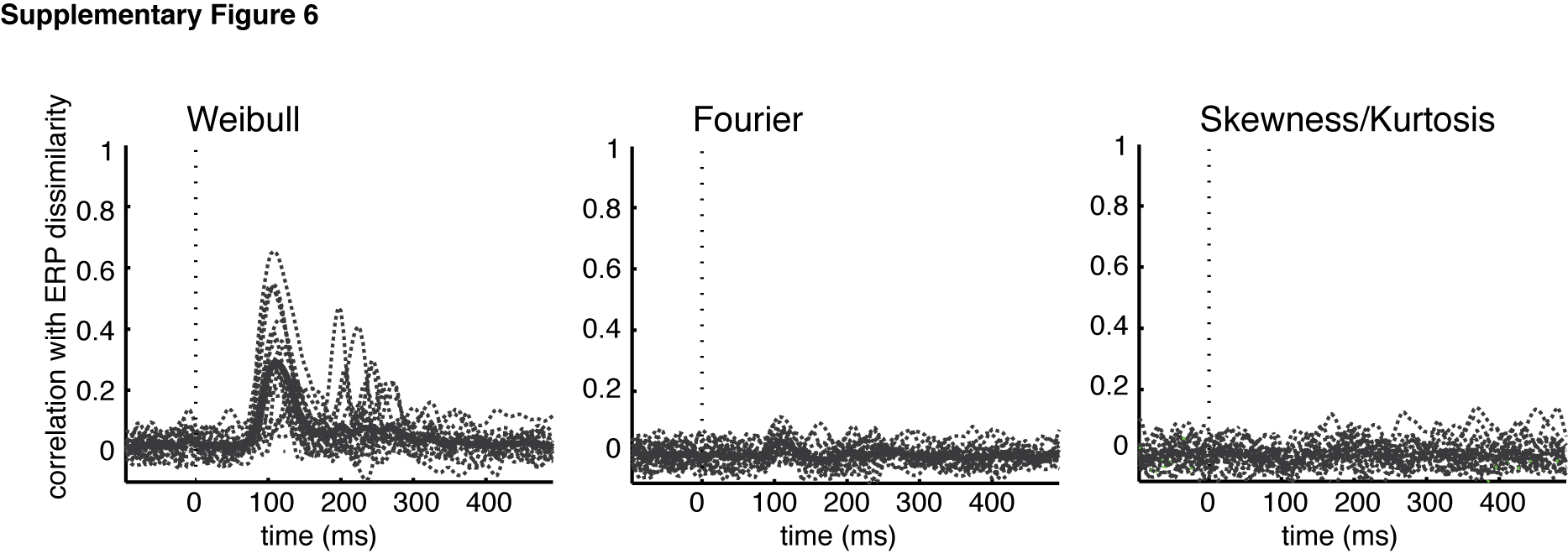

Supplement: Figure S6 — Single-subject correlations of dissimilarity matrices (RDMs) of ERPs with distance matrices based on the three sets of contrast statistics: (A), Weibull parameters, (B), Fourier parameters and (C), skewness and kurtosis. (TIF) [file pcbi.1002726.s006.tif]
